# Sleep and Sleep Disruption in Amyotrophic Lateral Sclerosis

**DOI:** 10.1007/s11910-020-01047-1

**Published:** 2020-05-27

**Authors:** Matthias Boentert

**Affiliations:** 1 Department of Neurology with Institute of Translational Neurology, University Hospital Münster (UKM), Albert-Schweitzer-Campus 1, Building A1, 48149 Münster, Germany; 2 Department of Medicine, UKM Marienhospital Steinfurt, Steinfurt, Germany

**Keywords:** Amyotrophic lateral sclerosis, Sleep, Sleep-disordered breathing, Insomnia

## Abstract

**Purpose of Review:**

In amyotrophic lateral sclerosis (ALS), sleep disruption is frequently present and substantially adds to disease burden. This review aims to summarize current knowledge on causes, pathophysiology, and treatment of sleep disturbances in ALS.

**Recent Findings:**

Motor neuron degeneration and muscle weakness may lead to muscle cramps, pain, spasticity, immobilization, restless legs, sleep-disordered breathing, and difficulties to clear secretions. Furthermore, existential fears and depression may promote insomnia. Sleep-disordered breathing, and nocturnal hypoventilation in particular, requires ventilatory support which meaningfully prolongs survival and improves health-related quality of life albeit respiratory failure is inevitable. Early indication for non-invasive ventilation can be achieved by inclusion of capnometry in diagnostic sleep studies.

**Summary:**

Sleep disruption is extremely common in ALS and may arise from different etiologies. The absence of causative therapeutic options for ALS underlines the importance of symptomatic and palliative treatment strategies that acknowledge sleep-related complaints.

## Introduction

Amyotrophic lateral sclerosis (ALS) is a neurodegenerative disorder characterized by progressive loss of upper and lower motor neurons. Consecutively, both spasticity and hyperreflexia may coexist with fasciculations and muscle atrophy, the latter resulting in skeletal muscle weakness virtually affecting all muscle groups. In bulbar-onset ALS, dysarthria and dysphagia usually predominate throughout the disease course. Spinal-onset subtypes of ALS arise from progressive loss of anterior horn cells which supply trunk and limb muscles [1, 2••]. Prevalence of ALS ranges from 5 to 8 per 100,000, and disease onset peaks between 50 and 70 years of age [3–6]. Lifetime risk for ALS has been reported to be 1:400 for women and 1:300 for men [7, 8].

Much is known about the molecular pathology of ALS and the genetic background of familial ALS subtypes [9] but no causative therapies have been developed to date. Only two compounds have been approved, including riluzole [10–12] and edavarone [13, 14], which both show disease-modifying effects but do not stop disease progression. Thus, muscle weakness is still inevitable and eventually leads to tetraplegia, dysarthria, swallowing dysfunction, and chronic hypercapnic respiratory failure [9, 15••]. Median survival has been reported to be 2.5–3.5 years after symptom onset and 1.5–2.5 years following diagnosis [16–18]. Chronic respiratory failure and its sequelae limit life span most and are the major cause of premature death in patients with ALS [19, 20]. ALS severely impacts activities of daily life and health-related quality of life for both patients and caregivers [21–24]. Since causative therapies are unavailable, symptom control is the hallmark of treatment, and a systematic approach to distinct disease aspects is recommended [15••].

Reduction of sleep quality substantially contributes to physical and mental health in patients with ALS [25–30]. Subsequently, sleep disturbances further increase the individual burden of disease. This review article aims to systematically outline sleep characteristics, sleep-related symptoms, and causes of sleep disruption in patients with ALS. Furthermore, it will focus not only on respiratory muscle weakness leading to sleep-related hypoventilation and chronic hypercapnic respiratory failure, but will also discuss “non-fatal” conditions which may also disrupt sleep and considerably impair health-related quality of life.

## Motor Symptoms of ALS and Sleep

Virtually, all motor symptoms of ALS may directly affect sleep quality, including fasciculations, muscle cramps, immobilization, and even restless legs syndrome (RLS). Furthermore, impaired swallowing function, if present, puts patients at risk of sialorrhea, recurrent choking, and aspiration of saliva.

Muscle fasciculations have been reported to cause sleep disturbances in some patients with ALS [31]. In addition, recurrent muscle cramps may occur, mainly affecting lower limb muscles and often exacerbating during the night. The International Classification of Sleep Disorders (ICSD-3 [32]) refers to nocturnal leg muscle cramps which are often painful, or inconvenient at least, and have been reported in patients with spinal-onset ALS, in particular [28, 33]. Electrophysiologically, muscle cramps reflect spontaneous discharges of motor units at a much higher frequency (> 300 Hz) than with voluntary contraction [34]. Active stretching may help ending these discharges but may be hampered in patients in whom significant limb weakness is present.

Symptomatic treatment of leg muscle cramps includes sufficient fluid intake, correction of electrolyte imbalances and, if acceptable, cessation of any causative medications. Mexiletine 150 mg twice daily has recently been reported to alleviate ALS-related muscle cramps in a randomized controlled trial [35]. Baclofen and other compounds did not show significant effects on muscle cramps in patients with ALS [36]. Quinidine (200–300 mg once to twice daily) has been evaluated in numerous other neurological conditions, showing reduction of both cramp frequency and intensity [37] but long-term use may be associated with severe thrombocytopenia, cinchonism, and myocardial toxicity [38].

As motor function gets worse, patients may have difficulties to change position in bed. Systematic studies on this issue are lacking, but its potential to severely impair sleep quality is obvious [29]. Furthermore, immobilization renders patients more dependent on caregivers whose intervention is frequently needed for both pain relief and prevention of skin lesions [39, 40].

Diagnostic criteria for RLS include an urge to move the legs (with unpleasant or painful sensations in the lower limbs), symptom onset and exacerbation only during rest or inactivity, circadian occurrence and enhancement of symptoms in the evening or during the night, and symptom relief during voluntary movement such as walking or stretching [32]. Symptom burden may vary inter-individually but profound sleep disturbances are common [41]. Prevalence of RLS in patients with ALS has been investigated in only few studies, all of them showing that RLS is significantly more frequent in ALS patients than in the general population [42–44]. Immobilization itself may put ALS patients at a greater risk to experience RLS which is reflected by the observation that RLS severity seems to be associated with overall neurological handicap in affected patients [42]. Mild sensory neuropathy, or even small fiber neuropathy, which have both been described in ALS may also contribute to RLS in this condition [45–47].

Symptoms of RLS in patients with ALS should be actively asked for, and pharmacological treatment strategies should follow standard recommendations regarding iron supplementation (if iron deficiency is present according to serum ferritin levels) or the use of dopaminergic agents, α2δ ligands, or opioids [48].

Periodic limb movements in sleep (PLM) have only rarely been evaluated in patients with ALS. Previous reports on PLM prevalence and event counts are inconclusive [49, 50], and it is still unclear whether PLM impact objective sleep in ALS. Own unpublished data suggest that the PLM index is increased in a large subset of patients but does not translate into associated arousals from sleep [51•]. A recent study postulated PLM disorder to be present in almost one-third of ALS patients but ICSD-3 diagnostic criteria were possibly not fully met, and association with sleep arousals was not reported [52]. Pathophysiologically, it may be hypothesized that in ALS, PLM reflect spinal cord disinhibition due to degeneration of descending central pathways [53].

## Nocturnal Pain in ALS

Pain and sleep quality are closely related: pain disrupts sleep and impaired sleep is known to worsen pain [54, 55]. Nocturnal pain in patients with ALS may directly result from immobilization and inability to change position in bed. Furthermore, intermittent muscle cramps or spasticity may induce pain, and muscle atrophy may enhance pressure load to bones and joints. A subset of patients with ALS may also experience neuropathic pain which is often not clearly localized [56, 57•] and may be attributable to small fiber neuropathy which has been described in up to 75% [45]. Finally, few patients report a third type of pain which is diffuse, non-neuropathic, independent of clear triggers, and possibly related to central sensitization of nociceptive pathways [58, 59]. Nocturnal pain in patients with ALS has not been specifically investigated in clinical studies [60]. Treatment strategies in patients with ALS usually follow clinical considerations rather than scientific evidence. Clearly nociceptive pain may require adequate preventive measures and non-steroidal anti-inflammatory drugs. Opioids may be added for otherwise refractory pain or if dyspnea is also present. Cannabis compounds may also be prescribed, having also sedating, anxiolytic, and appetite-enhancing effects. For alleviation of neuropathic pain α2δ ligands or antidepressants should be considered, and spasticity may require central muscle relaxants. To conclude, patients with ALS frequently report pain of different origin, potentially disrupting sleep. Since chronic pain co-determines subjective quality of life and suicidality especially in later stages of ALS, it should be considered a major target of symptomatic therapy [61].

## Sleep-Related Breathing Disorders

In patients with ALS, sleep-related breathing disorders mainly comprise nocturnal hypoventilation (NH) and obstructive sleep apnea (OSA). Central sleep apnea has only rarely been reported [62, 63]. Prevalence of OSA is particularly increased in male patients and, interestingly, in patients with spinal (or non-bulbar) onset of symptoms [51, 63]. In bulbar-onset ALS, OSA might be less likely to occur because atrophy of the tongue prevents pharyngeal collapse. The presence of OSA prior to initiation of ventilatory support has been related to shorter survival but often coincides with respiratory muscle weakness which mainly determines further disease progression [51•, 64, 65]. Sleep-related hypoventilation mainly results from phrenic nerve degeneration and diaphragm weakness leading to carbon dioxide accumulation during rapid eye movement (REM) sleep first [66]. With disease progression, hypercapnia spreads to non-REM sleep stages, and finally, daytime hypercapnia defines chronic hypercapnic respiratory failure. Associated symptoms include sleep disruption, daytime sleepiness, morning headache, and dyspnea in various situations, i.e., during sleep, when supine, on exertion, or at rest. In order to prevent orthopnea patients with advanced diaphragm weakness may adopt a sitting position in bed. Several studies have shown that sleep-disordered breathing impairs both continuity and composition of sleep in ALS patients. Apneas, hypopneas, and impaired gas exchange are associated with reduced sleep efficiency, frequent sleep stage changes, arousals from sleep, and reduction of N3 or REM sleep [51•, 62, 67–71]. For detection of nocturnal hypoventilation, pulse oximetry has widely been used but transcutaneous capnometry has proven to be superior since peripheral oxygen saturation may be normal in one-third of patients with sleep-related hypercapnia [51•, 72]. Thus, transcutaneous capnometry is indispensable for evaluation of sleep-related breathing in ALS in order to warrant that diagnosis of nocturnal hypoventilation and initiation of non-invasive ventilation (NIV) can be achieved early. Notably, nocturnal hypercapnia may be present long before patients report sleep disturbances or dyspnea [73]. For identification of patients who should be referred for sleep studies, predictors of nocturnal hypoventilation and impending respiratory failure are desirable. Bedside tests of respiratory muscle strength include forced vital capacity (FVC), maximum inspiratory pressure (MIP), and sniff nasal inspiratory pressure (SNIP), all being predictors of survival and disease progression [74–76]. For prediction of nocturnal hypoventilation and NIV indication, SNIP might be most suitable in ALS [77•, 78] but FVC and MIP should be regularly monitored because current guidelines for initiation of NIV in patients with neuromuscular disease rely on these measures [79]. In two recent studies, several factors have been found to be associated with early onset and rapid deterioration of respiratory insufficiency, including higher age at diagnosis, longer diagnostic delay, lower FVC and body mass index, bulbar onset of symptoms, and a lower ALS functional rating scale (ALSFRS-R) total score or dyspnea subscore, respectively [80, 81•]. Another study showed that nocturnal oxygen desaturations are also associated with respiratory failure and worse prognosis [82].

A plethora of retrospective studies and one randomized trial confirmed that NIV prolongs survival in patients with ALS [17, 83–95]. Furthermore, recent work suggests that effects on life expectancy are stronger when NIV is started early, i.e., in patients with only mild FVC reduction [96••]. Long-term NIV has the potential to sustainably improve sleep quality and quality of life although ventilator dependency progressively increases over time [67, 69, 97–99]. As prerequisites of these effects, optimal mask fitting, adequate titration of respirator settings, and education of patients and caregivers are all crucial. Treatment adherence is strongly influenced by mask selection and fitting. Nasal interfaces are often less uncomfortable but may be insufficient if mouth leaks occur, and oronasal masks may be preferred [100]. However, the latter may promote treatment-induced upper airway obstruction, most likely by dorsal displacement of the tongue, which requires adjustment of pressure settings and end-expiratory pressure, in particular [101]. Furthermore, upper airway obstruction may occur independently of the type of mask in patients with pseudobulbar palsy, in particular. Upper motor neuron dysfunction at the bulbar level has recently been shown to put affected patients at risk of intermittent glottic closure as well as decreased respiratory drive rendering NIV transiently ineffective when both coincide [102•].

Volume support ventilation has been proven to be especially effective with regard to gas exchange and symptom alleviation but may promote patient-ventilator dyssynchrony as reflected by ineffective or inappropriate triggering, auto-triggering, or flow dyssynchrony [103]. Follow-up sleep studies should comprise poly(somno)graphy and capnometry because prognosis may be worse when normocapnia, normoxia, and normalization of the apnea hypopnea index are not reliably achieved by NIV [104, 105, 106•].

Whereas older studies did not show that NIV specifically prolongs life span in patients with bulbar ALS [88], recent work suggests the opposite [107]. Furthermore, NIV improves quality of life and sleep quality in this subgroup [67, 69]. However, dysphagia and mucus retention have to be taken in account with particular care if bulbar disease is present. In general, management of secretions is highly important for all ALS patients with respiratory muscle weakness, including manually assisted coughing or mechanical cough assistance [108, 109].

## Behavioral Abnormalities During Sleep

Behavioral abnormalities during sleep, or parasomnias, include movements or behaviors which either arise from sleep or occur on falling asleep or awakening. Parasomnias are characterized by partial arousals from either non-REM or REM sleep [32]. No evidence is available suggesting that NREM parasomnias (i.e., sleepwalking, pavor nocturnus, or confusional arousals) specifically occur in patients with ALS. REM behavioral disorder (RBD) is characterized by persistent phasic or tonic muscle activation during REM sleep [110]. Patients with manifest RBD show dream-enacting movements and vocalizations possibly leading to injuries, bed falls, or aggressive behaviors involving the bed partner [32]. RBD is mainly associated with neurodegenerative disorders and synucleinopathies in particular [111, 112] but may not exclusively relate to α-synuclein pathology since it has been reported in anti-IgLON5 disease, frontotemporal dementia, and certain rare forms of familial ALS [113–116]. In sporadic ALS, RBD or REM sleep without atonia may be found in a small number of patients possibly indicating that neurodegeneration involves REM sleep regulatory pathways [50]. Further systematic studies are desirable to elucidate both the pathophysiology and clinical relevance of this observation.

## Conclusions and Future Perspectives

Sleep disturbances are common in patients with ALS and significantly add to the individual burden of disease. Major causes comprise sleep-disordered breathing, immobilization, muscle cramps, RLS, and other painful or unpleasant sensations which are mostly attributable to progressive impairment of motor function. Sleep-related symptoms have to be actively asked for, and treatable conditions should be treated in order to improve well-being and quality of life. Apart from clearly physical causes of sleep disruption, psychological aspects such as fear, depression, or recurrent grief and despair have to be acknowledged and may significantly contribute to insomnia in patients with ALS [117, 118]. With regard to disease-specific characteristics of sleep composition and regulation, future research will extend preliminary evidence from recent studies suggesting that motor control during REM sleep [50], sympathovagal balance [119], and central respiratory control [102•] may all be altered in patients with ALS, possibly contributing to sleep disruption already in early stages of the disease.

## Abbreviations

ALS: Amyotrophic lateral sclerosis
ALS-FRS-R: ALS functional rating scale (revised)
FVC: Forced vital capacity
MIP: Maximum inspiratory pressure
NIV: Non-invasive ventilation
OSA: Obstructive sleep apnea
PLM: Periodic leg movements
RBD: REM sleep behavioral disorder
REM: Rapid eye movement
RLS: Restless legs syndrome
SNIP: Sniff nasal inspiratory pressure
